# Nanobody Technology: A Versatile Toolkit for Microscopic Imaging, Protein–Protein Interaction Analysis, and Protein Function Exploration

**DOI:** 10.3389/fimmu.2017.00771

**Published:** 2017-07-04

**Authors:** Els Beghein, Jan Gettemans

**Affiliations:** ^1^Nanobody Laboratory, Department of Biochemistry, Faculty of Medicine and Health Sciences, Ghent University, Ghent, Belgium

**Keywords:** nanobody, VHH, single-domain antibody, engineering, super-resolution microscopy, protein–protein interactions, targeted protein degradation, fundamental research

## Abstract

Over the last two decades, nanobodies or single-domain antibodies have found their way in research, diagnostics, and therapy. These antigen-binding fragments, derived from Camelid heavy chain only antibodies, possess remarkable characteristics that favor their use over conventional antibodies or fragments thereof, in selected areas of research. In this review, we assess the current status of nanobodies as research tools in diverse aspects of fundamental research. We discuss the use of nanobodies as detection reagents in fluorescence microscopy and focus on recent advances in super-resolution microscopy. Second, application of nanobody technology in investigating protein–protein interactions is reviewed, with emphasis on possible uses in mass spectrometry. Finally, we discuss the potential value of nanobodies in studying protein function, and we focus on their recently reported application in targeted protein degradation. Throughout the review, we highlight state-of-the-art engineering strategies that could expand nanobody versatility and we suggest future applications of the technology in the selected areas of fundamental research.

## Introduction

**Figure 1:**
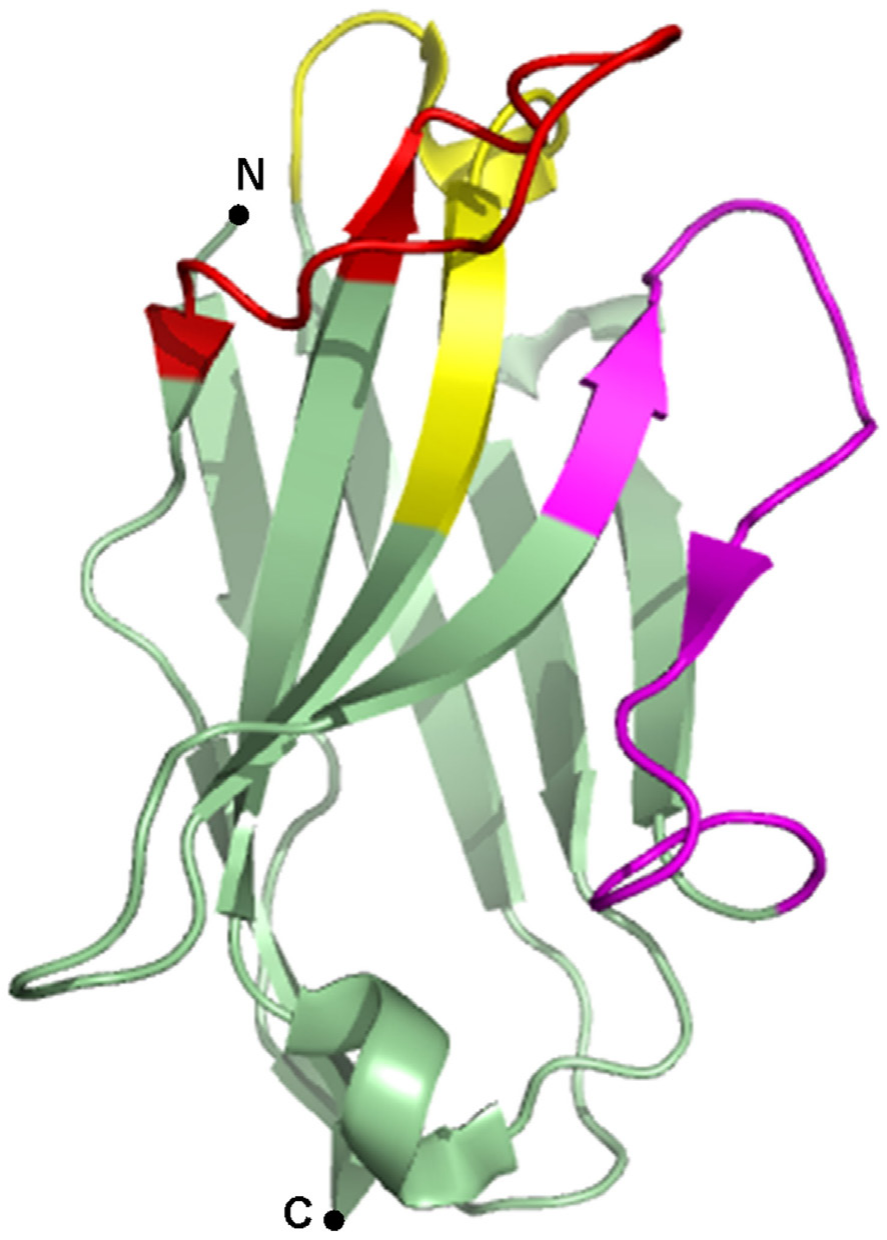
Crystal structure of a gelsolin nanobody. A nanobody is typically composed of three complementarity-determining regions (CDRs), alternated with four framework regions (FRs). CDR1 is depicted in yellow, CDR2 in magenta, CDR3 in red, and the FRs are depicted in green. Image of PDB ID 2X1O (5) created with PyMOL.

Since the discovery of heavy chain only antibodies (HcAbs) in 1993 by the Hamers-Casterman’s group (1), the use of their antigen binding fragments or nanobodies in research, diagnostics, and therapy has evolved at an incredible pace. HcAbs are unique IgGs that are found in sera of *Camelidae*. These antibodies are devoid of the light chain and lack the first constant domain. Consequently, the antigen-binding fragment of HcAbs is solely composed of a single variable domain, referred to as VHH (variable domain of the heavy chain of HcAbs), single-domain antibody or nanobody, which is only ~15 kDa in size. The variable domains of conventional IgGs and HcAbs comprise three complementarity-determining regions (CDRs) that constitute the paratope of the antibody (Figure 1). As nanobodies lack the variable domain of the light chain, they only contain three instead of six CDRs. These CDRs are organized in three loops, separated by more conserved framework regions (FRs) and cluster at the N-terminal side of the nanobody. In order to provide an adequate antigen-interacting surface of 600–800 Å^2^, nanobodies have longer CDR1 and CDR3 loops than VHs (variable domain of the heavy chain) of conventional antibodies, resulting in similar binding affinities. In dromedary nanobodies, these long loops are often connected by a disulfide bond that restricts their flexibility and consequently, favors antigen binding. Normally, FR2 region of VHs contains highly conserved hydrophobic amino acids participating in the interaction with the VL (variable domain of the light chain). As this region is water-exposed in nanobodies, the hydrophobic amino acids are substituted by hydrophilic residues, which reduce the likelihood for aggregation. This explains the high solubility of nanobodies (2–4).

Nanobodies are thus endowed with favorable characteristics in terms of size, solubility, and affinity. Furthermore, nanobodies can easily be produced recombinantly in bacteria, yeast, plants, and mammalian cell lines (3, 4). When expressed in eukaryotic cells as intrabodies, nanobodies accurately bind and trace their target as they normally do not appear to suffer from the reducing intracellular environment. Nanobodies can easily be equipped with a customized tag (e.g., fluorescence, affinity, epitope tag, etc.) without losing their affinity or stability (6–9). Moreover, nanobodies feature a convex paratope and can, therefore, but also due to their small size, bind hidden epitopes in small cavities (e.g., active site of enzymes) (10, 11). They mainly bind conformational epitopes (7, 10, 12), but nanobodies recognizing linear epitopes have also been reported (5, 13, 14). These unique biochemical and biophysical properties of nanobodies purportedly render them superior to conventional antibodies or antibody fragments, and make them ideally suited for a myriad of biotechnological applications.

Despite the aforementioned benefits of the nanobody technology, still some drawbacks need to be overcome. First of all, unmodified nanobodies are not able to traverse the cell membrane. Using nanobodies in research thus requires transfection or transduction in cells, or requires the use of transgenic animals. However, several research groups are looking into this issue. Possible solutions are coupling the nanobodies to a cell-penetrating peptide (penetratin) (15) or exploiting the *Escherichia coli* type III protein-secretion system (T3SS) (16, 17). Second, although quite exceptional, nanobodies can lose their functionality when expressed intracellularly (7). A third and perhaps major stumbling block, is the fact that nanobody production (animal housing, immunization, library construction, and phage panning) is equivalent to monoclonal antibody production, CRISPS/Cas9 mouse knockouts, and hence relatively expensive.

In this review, we assess the current status of nanobodies as research tools in diverse facets of fundamental research (microscopy, protein–protein interactions and protein function). Moreover, we focus on the adaptability of nanobodies, or how engineering can expand their versatility, and we discuss future opportunities given the current know-how. As the use of nanobodies in diagnostics and therapy does not fall within the scope of this paper, we refer the reader to some excellent recent reviews (18, 19).

## Nanobodies Used as Research Tool in Microscopy

### Primary Detection Reagents in Fluorescence Microscopy

Several studies confirmed the usefulness of nanobodies as equivalent detection surrogates for antibodies in immunocytochemistry (Table 1). de Bruin and coworkers generated and characterized anti-Vγ9 and anti-Vδ2-T cell receptor-directed nanobodies that could successfully be used as primary detection reagents for Vγ9Vδ2-T cells in immunocytochemistry (20). Bound nanobody was detected using a secondary anti-nanobody antibody, followed by a tertiary Alexa Fluor 488-conjugated antibody (20). To shorten staining procedure, Jullien and colleagues mixed their HA-tagged histon H2A-H2B nanobody (chromatibody) with an anti-HA antibody for primary staining (9). Using a tertiary fluorescently labeled antibody, chromatin-specific staining was observed in human HCT116 cells and even in organisms evolutionarily distant from mammals (9). Peyrassol and colleagues developed His-tagged ChemR23 G-protein-coupled receptor (GPCR) nanobodies and tested their binding specificity by immunostaining on fixed CHO cells (21). Visualization was performed by using a fluorescently labeled anti-His secondary antibody, hence avoiding the use of a tertiary antibody (21).

**Table 1:** Overview of the different nanobody-based applications in microscopy.

	Application	Strategy	Specifics	Reference
Microscopy	Primary detection reagents in fluorescence microscopy	Indirect immunocytochemistry		(9, 20, 21)
		Direct immunocytochemistry		(8, 13)

	Primary detection reagents in super-resolution microscopy	Anti-GFP and anti-RFP nanobodies	*N*-hydroxysuccinimide *(NHS)* ester-labeling	(22–25)
		Nanobodies targeting endogenous protein	*N*-hydroxysuccinimide *(NHS)* ester-labeling	(26)
			Cysteine-maleimide-labeling	(27, 28)
			Sortase A-labeling	(29)
			Furan-labeling	(30)

	Intracellular nanobodies as microscopic tracers			(6–9, 31–33)

*Each application corresponds to the different sections in the main text and the strategies match the different paragraphs therein, which are whether or not further specified*.

Equipping nanobodies with organic fluorescent dye bypasses the use of a secondary and/or tertiary fluorescently labeled antibody and thus makes the staining procedure cheaper and less elaborate. Braun and colleagues characterized an anti-β-catenin nanobody, referred to as BC2-VHH, which recognizes a linear epitope of only 12 amino acids with low nanomolar affinity (13). Coupling this nanobody to the organic dyes Alexa Fluor 488 or ATTO 647 by means of *N*-hydroxysuccinimide ester-labeling (see Primary Detection Reagents in Super-Resolution Microscopy) endows it with the capability to visualize BC2-tagged fusion proteins directly (13). Accordingly, Maier and colleagues provided their vimentin nanobodies with a fluorescent ATTO 488 tag (8). Binding specificity was examined in different cell lines (8). Of note, the mentioned VB6 vimentin nanobody is not a genuine nanobody, but a variable domain derived from a conventional antibody.

### Primary Detection Reagents in Super-Resolution Microscopy

Diffraction of light limits the resolution of conventional fluorescence microscopy to about 200–300 nm in the lateral and 500–700 nm in the axial direction, leaving many subcellular structures too small to be observed in detail. Several variants on fluorescence microscopy, such as confocal or multiphoton microscopy, only enhance resolution moderately. Ground-breaking progress was made in the 1990s, when a number of super-resolution techniques arose that achieve resolutions far beyond the limit of diffraction, for instance, STED (stimulated emission depletion), STORM (stochastic optical reconstruction microscopy), or PALM (photoactivated localization microscopy) (34, 35). Theoretically, the resolution of these techniques can reach molecular scale. In practice however, resolution is limited by a combination of intrinsic optical properties and sample specific factors. An example of the latter is the size of the fluorescent labels, which become significant at high resolution (35). Using indirect immunochemistry for protein detection, the primary and secondary antibody increases the apparent size of the visualized structure or introduces a localization bias of 10–20 nm (22, 36, 37). Reducing the distance between the antigen and fluorescent label (linkage error) can be achieved by directly coupling an organic dye molecule to a peptide sequence, which is genetically fused to the protein of interest (23, 38, 39). For instance, coupling proteins to a 15 amino acid acceptor peptide tag, allows enzymatic biotinylation and consequent visualization of the protein with fluorophore-labeled monomeric streptavidin (23). Nevertheless, in some experiments, genetic engineering or overexpression is not appropriate (e.g., in case of human samples, peptide interfering with protein interactions or due to lack of time). In these cases, large linkage error can be tackled by direct immunofluorescence, using fluorescently labeled conventional antibodies (40).

Recently, the use of labeled nanobodies as nanoscale detection tools has emerged (Table 1), since nanobodies are significantly smaller than antibodies. Several publications describe the use of anti-GFP and anti-RFP nanobodies for super-resolution microscopy. These nanobodies target genetically encoded fluorescent fusion proteins and are equipped with a strong organic dye, usually coupled to the nanobody by means of *N*-hydroxysuccinimide ester-labeling (see later in this section) (22–24). The first use of this technology was reported by Ries and coworkers (22). They labeled individual microtubules in fixed Ptk2 cells stably expressing tubulin-YFP. The acquired resolution of 26.9 ± 3.7 nm is compatible with a microtubule diameter of 25 nm and is considerably smaller than what was achieved with indirect immunochemistry using conventional antibodies (±45 nm). Moreover, these nanobodies showed also to be valuable tools for high resolution live imaging and dual-color microscopy (22). Accordingly, Chamma and coworkers used GFP nanobodies to live-label synaptogenic adhesion protein neurexin-1β and to image transsynaptic contacts in neurons in a dual-color setup (23). GFP and RFP nanobodies can also be used to study nuclear pore complex (NPC) and caveolae ultrastructure in detail. Unlike indirect antibody immunochemistry, nanobody staining resulted in a far better approximation of the actual dimensions of both structures (24, 25).

Using GFP or RFP nanobodies as detection tool has some advantages. First, high-affinity GFP and RFP nanobodies are commercially available (41), which makes it possible to visualize virtually every protein; even those for which no specific targeting moiety is available. Moreover, it allows comparable and quantitative labeling between different proteins. This method can also be used to image GFP-tagged proteins from GFP-fusion libraries in high throughput (22). Nevertheless, experiments sometimes require visualization of endogenous protein or overexpression of fusion protein is not appropriate (see above). In these cases, endogenous target-specific nanobodies can be used. Excluding the GFP/RFP-tag for detection practically minimizes linkage error to the length of a nanobody, which is 2–4 nm.

**Figure 2:**
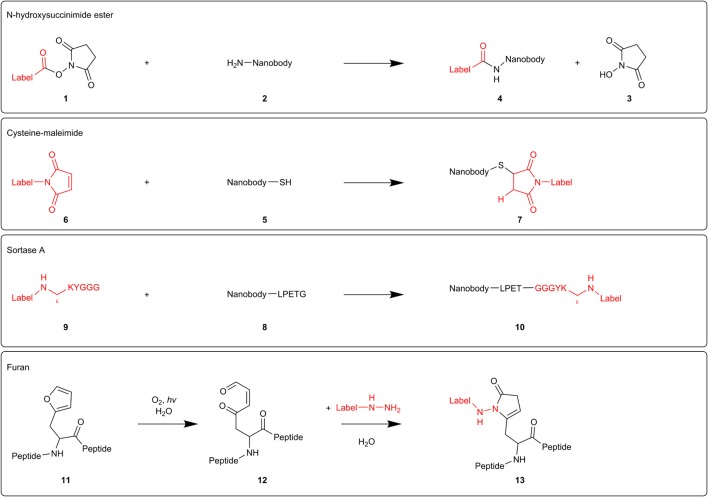
Mechanisms of different nanobody-labeling strategies for super-resolution microscopy. *N*-hydroxysuccinimide ester-labeling **1** (top) randomly labels primary amines **2** in the nanobody. The other techniques mentioned (cysteine-maleimide, Sortase A, and furan technology) site-specifically label introduced tags (respectively, cysteine **5**, sortag or LPETG **8**, and furylalanine **11**).

In fact, every nanobody compatible with immunostaining can be used for super-resolution microscopy. Different labeling techniques have been reported. *N*-hydroxysuccinimide (NHS) ester-labeling **1** of primary amines **2** (R-NH_2_) is the most widespread labeling strategy (Figure 2). NHS ester derivatives of various fluorescent probes are commercially available. The carbonyl carbon of the NHS ester reacts with primary amines in the nanobody, thereby releasing NHS **3** and crosslinking the nanobody with the organic dye **4**. Accordingly, Mikhaylova and coworkers conjugated their anti-β-tubulin nanobodies with Alexa Fluor 647 (26). Applying these nanobodies in super-resolution microscopy, they succeeded in resolving individual microtubules, both *in vitro* and in fixed cells. Furthermore, for densely packed microtubules with a 25-nm lattice-to-lattice spacing, the resolving power of the nanobodies was 2.5-fold and 10-fold higher than primary and primary–secondary antibody labelings, respectively (26).

NHS ester-labeling can, however, abolish or reduce antigen-recognition of the nanobody if the paratope contains primary amines that become labeled. Equipping the nanobodies with a C-terminal oligo-lysine stretch might divert NHS-labeling from intrinsic nanobody lysine residues (24). However, modification of multiple lysines can create hydrophobic patches that increase unspecific binding and thus background staining (27). Several research groups, therefore, attempt to develop a generic site-specific conjugation method. These techniques make it possible to control where and how many labels will be added, resulting in a homogeneous nanobody population.

Massa and coworkers labeled anti-HER2 nanobody using the cysteine-maleimide strategy (28) (Figure 2). They introduced a unique place for conjugation by equipping the nanobody with a C-terminal cysteine **5**, spaced by a rigid 14 amino acid linker from the nanobody sequence. This linker presumably prevents the added cysteine from interfering with correct folding of the dromedary nanobody interloop disulfide bond. When adding a bifunctional maleimide-label **6**, the maleimide double bond reacts with the cysteine thiol group, generating a stable carbon-sulfur bond **7**. However, this derivatization strategy resulted in a severe reduction in production yields and triggered extensive dimerization of the nanobodies at the introduced C-terminal cysteine, necessitating an additional reduction step. In order to safeguard intradomain disulfide bonds, the reducing agent needs to be titrated carefully (28). Pleiner and coworkers used the cysteine-maleimide labeling in order to visualize individual NPC proteins or nucleoporins (27). They mutated one or more solvent-exposed small residues (framework glycine, serine, or alanine) to cysteines or introduced an N or C-terminal cysteine in GFP nanobody and several nucleoporin nanobodies. These cysteines were subsequently crosslinked with maleimide-Alexa Fluor 647/488. The conjugation reaction was performed at 0°C in order to protect the intradomain cysteines. In confocal laser scanning microscopy, all nanobodies produced a bright punctuate nuclear rim staining against a very low background, even when there was only one dye molecule per nanobody. Strikingly, cysteine-maleimide-labeled nanobodies performed far better than their NHS ester-labeled counterparts in terms of specificity. The nucleoporin nanobodies also performed excellent in super-resolution microscopy, providing very detailed views of individual NPC proteins (27).

Recently, two novel derivatization techniques were reported that hold great promise for future nanobody-labeling with organic dyes. First, researchers exploited a transpeptidase Sortase A (SrtA) derived from *Staphylococcus aureus* to label an anti-HER2 nanobody with the fluorescent dye Cy5 (Figure 2). Therefore, nanobodies were provided with a C-terminal SrtA recognition motif or sortag (LPETG) **8**, and Cy5 was coupled to the pentapeptide GGGYK *via* the side chain ε-amine of the lysine residue **9**. SrtA catalyzes the formation of a new peptide bond between the threonine of the sortag and the glycine of the pentapeptide, hence generating a stable bond between nanobody and fluorescent probe **10**. The labeled HER2 nanobody performed excellent in fluorescence reflectance imaging of HER2-positive tumors in mice (29).

The furan crosslinking technology comprises a second potential derivatization approach (Figure 2). Albeit not shown for nanobodies yet, researches already successfully labeled thymosin β4 peptides with different fluorescent dyes using this technique. Briefly, a furylalanine building block **11** was incorporated into thymosin β4 peptide. Photooxygenation of the furan moiety results in the formation of a 4-oxo-enal moiety **12**. Subsequent addition of a NH_2_NH-coupled label, transforms the furan-containing peptides into pyrrolidinone-based fluorescent probes **13** (30). As super-resolution microscopy techniques can be exploited to their full potential by using nanobodies as detection tool, more site-specific conjugation methods will undoubtedly emerge in the near future.

### Intracellular Nanobodies As Microscopic Tracers

Target visualization can also be achieved by intracellular expression of fluorescently labeled nanobodies (chromobodies) or nanobodies equipped with an epitope tag that allows antibody detection (Table 1). These intrabodies typically do not interfere with protein function and allow visualization of the endogenous target. Overexpression of (fluorescent) fusion protein is thus no longer needed, which frequently induces artificial changes in cell behavior (8, 9, 31) or results in a false representation of protein dynamics (26). Our lab generated a nanobody against survivin, a protein that exerts key roles during mitosis (7). The survivin nanobody was equipped with a V5-tag, enabeling immunocytochemical detection using an anti-V5 antibody. The nanobody accurately tracks its target during different phases of mitosis and moreover, it detects different surviving subpopulations that are indiscernible for certain commercially antibodies (7). Similarly, intracellular expression of EGFP-tagged nuclear transport factor 2 (NTF2) nanobodies uncovered a new location of NTF2 at the centrosome (6). Maier and colleagues on the other hand, expressed a set of EGFP-labeled vimentin chromobodies in HeLa cells and compared their localization pattern to a canonical anti-vimentin antibody staining (8). As such, they could identify in an early screen which nanobodies are genuine vimentin binders (8). Accordingly, Van Overbeke and coworkers validated binding specificity of endoplasmic reticulum-directed gelsolin nanobodies by immunocytochemistry (32). Colocalization between plasma gelsolin and the V5-tagged nanobodies confirmed proper nanobody binding (32). Fluorescent nanobodies are also excellent research tools for live imaging in cells and whole organisms. The aforementioned vimentin nanobodies were further utilized to monitor endogenous vimentin localization and dynamics in A549 lung cancer cells. In this cell-based chromobody model, it was possible to monitor dynamic changes of vimentin in real-time upon RNAi treatment or induction with TGF-β (8). Recently, similar high resolution spatiotemporal antigen tracking was reported using histon H2A-H2B (9), β-catenin (31), F-actin, and PCNA (33) nanobodies.

## Nanobodies Used as Research Tool to Identify Protein–Protein Interactions

### GFP-Targeting Nanobodies

**Figure 3:**
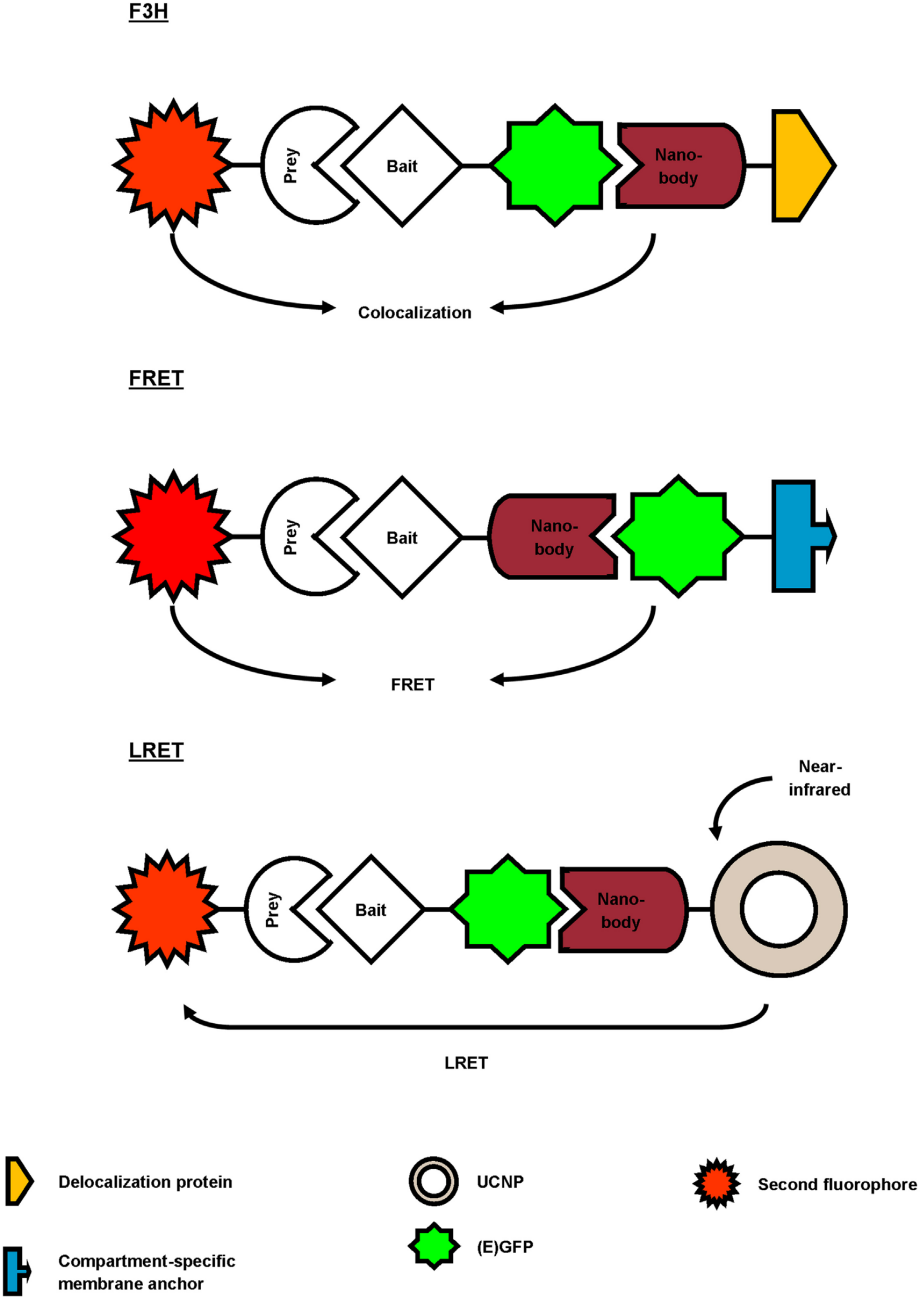
Overview of different reported approaches to investigate protein–protein interactions using a GFP-targeting nanobody. In the fluorescent-three-hybrid (F3H) strategy (top), the GFP nanobody delocalizes GFP-tagged bait toward a defined subcellular location. The nanobody can also be fused with bait protein and as such, relocalize bait toward GFP-tagged membrane protein (middle). Finally, the GFP nanobody can be used to deliver upconversion nanoparticles (UCNPs) toward GFP-tagged bait (bottom). Binding between GFP-bait and labeled prey is then validated by colocalization, Förster or lanthanide-based resonance energy transfer (FRET or LRET, respectively).

Several studies report the use of a GFP-targeting nanobody to study protein–protein interactions (Figure 3; Table 2). Herce and colleagues presented the fluorescent-three-hybrid (F3H) strategy as an alternative to the well-known yeast two-hybrid (Y2H) technique (42). They coupled GFP nanobody with a delocalization tag that redirects GFP-tagged bait protein and eventually mCherry-tagged prey toward a well-defined subcellular location. (Co-)localization of bait and prey can be visualized using fluorescence microscopy. Moreover, real-time imaging allows monitoring of the inhibition kinetics of interactions induced by drugs. The F3H approach was validated for delocalization to various subcellular compartments (Lac operator DNA sequence, chromocenters, nuclear lamina, and centrioles), for different cell types and species (baby hamster kidney, mouse myoblast C2C12, and human cervical carcinoma HeLa), emphasizing on the flexibility of the technique. F3H does not require specialized equipment (42). Moreover, this technique overcomes several important drawbacks of Y2H associated with the reporter system or the use of yeast as host (42, 43). To circumvent overexpression of fluorescent fusion proteins (bait and prey), one can use a nanobody that targets and delocalizes endogenous protein toward predetermined organelles. Potential interactors colocalize with the target protein, which can be visualized by post-fixation labeling (6). This approach does, however, not allow studying interaction or disruption kinetics.

**Table 2:** Overview of the different nanobody-based applications to identify protein–protein interactions.

	Application	Strategy	Specifics	Reference
Identify protein–protein interactions	GFP-targeting nanobodies	Fluorescent-three-hybrid		(42)
		Förster resonance energy transfer		(44)
		Lanthanide-based resonance energy transfer		(45)

	Nanobodies in mass spectrometry	Affinity-purification mass spectrometry *(AP-MS)*	Classical *AP-MS*	(27, 46)
			Footprinting	(6)
			Virotrap	(47)
			BioID proximity-labeling	(48)
		Organellar proteomics		(7)
		Hydrogen/deuterium exchange		(49–52)

*Each application corresponds to the different sections in the main text and the strategies match the different paragraphs therein, which are whether or not further specified*.

Künzl and coworkers studied vacuolar sorting in plants using a GFP nanobody sensor (44). Soluble proteins are sorted to the vacuole for degradation. Sorting relies on the activity of vacuolar sorting receptors (VSRs) that bind proteins by means of a luminal binding domain (LBD). However, not much was known about the exact locations (endoplasmic reticulum, Golgi, trans-Golgi network/early endosome or multivesicular late endosomes) at which VSRs bind or release their ligands. In order to investigate this, universal and compartment-specific VSR sensors were generated and expressed in tobacco mesophyll protoplasts. A full-functional VSR sensor consists of a LBD (bait)-equipped GFP nanobody and a GFP-tagged membrane marker protein. The latter fluorescently decorates the membrane of a specific compartment, depending on the chosen marker (e.g., GFP-calnexin for visualization of endoplasmic reticulum). A RFP-coupled model ligand (prey), containing a vacuolar sorting motif, was used to study compartment-specific interactions between LBD (bait) and ligand (prey). Upon coexpression and binding of the three constructs, the excited-state energy from GFP (membrane marker) is transferred to RFP (ligand), thereby reducing the fluorescence lifetime of GFP. This phenomenon, also called Förster resonance energy transfer (FRET), can be detected using fluorescence lifetime imaging and makes it possible to discern true binding from interaction-independent colocalization. FRET ceases at the cellular compartment when LBD (bait) releases its ligand (prey). As such, a novel pathway of vacuolar protein sorting in plants was postulated (44).

GFP nanobody has also been exploited as targeting moiety for upconversion nanoparticles (UCNPs) in lanthanide-based resonance energy transfer (LRET) imaging. In simple terms, lanthanide-doped UCNPs are able to convert two or more near-infrared photons into one UV/Vis photon. In its turn, this photon can sensitize a neighboring acceptor fluorophore. The UCNPs were functionalized with anti-GFP nanobody to target a bait EGFP-fusion protein. On the other hand, prey protein was fused to an acceptor fluorophore. Sensitized fluorescence upon LRET from the UCNPs can only be detected when bait and prey interact (in)directly. As proof-of-concept, the indirect interaction between mitochondrial outer membrane (MOM) proteins Tom20 and Tom7 was successfully visualized using UCNP LRET (45).

In the latter three studies, a high-affinity GFP nanobody was used to study protein–protein interactions in living cells, albeit combined with different techniques (F3H, FRET, or LRET). The nanobody was utilized as delocalization tool, thereby enriching bait and eventually prey at defined subcellular locations (F3H, FRET) (42, 44), or was used to target a reporter toward bait protein (LRET) (45). As such, the GFP nanobody emerges as a highly adaptable research tool to study protein–protein interactions.

### Nanobodies in Mass Spectrometry (MS) and Perspectives

Nanobodies are valuable tools for MS applications (Table 2). Recently, nanobodies have been used as an alternative for antibodies in classical affinity-purification mass spectrometry (AP-MS) to study protein complexes (27, 46). Their small size minimizes background binding and reduces the amount of tryptic peptides released from the affinity resin during on-bead digestion (53). Hypothetically, background binding in AP-MS can be further tackled by nanobody footprinting. In brief, by using nanobodies that target different epitopes in the same protein, true interaction partners may be displaced. This leaves a footprint; hence the name nanobody footprinting (6). Proteins that are shared among different nanobody-based APs either represent false positives or genuine binders that interact with the antigen at an epitope that is not recognized by the nanobodies. False positives can, in their turn, be significantly eliminated by using an appropriate control nanobody (e.g., GFP nanobody) for AP. This finally results in a (shorter) list of *bona fide* interaction partners. Combining this strategy with Virotrap, a lysis-free protein interaction analysis method, could improve the study of protein complexes, as lysis-sensitive protein complexes are preserved. Virotrap implies trapping a bait protein, together with its putative interaction partners, inside protective virus-like particles (VLPs) that bud from cells. Following antibody-based enrichment and lysis of the VLPs, protein complexes can be analyzed by Western blot or MS. Packing of bait in VLPs is achieved by expression of p55 HIV-1 GAG-bait fusion protein (e.g., expression of GAG-HRAS to detect the HRAS–RAF1 interaction) (47). Similarly, nanobody could be fused to a GAG protein and capture its target (and target interactors) in VLPs, thus avoiding overexpression of bait protein. Lysis-sensitive protein interactions can also be detected by using the BioID proximity-labeling strategy. This technique implies coupling bait to BirA*, a promiscuous biotin ligase that covalently attaches a biotin molecule to exposed lysine residues in proximate and interacting prey. All biotinylated prey is subsequently collected by means of streptavidin-AP and analyzed with MS. Consequently, the technique allows detection of weak and transient protein interactions that could be missed when using classical AP-MS (48). Combining BioID with nanobody footprinting could provide more details on the epitopes of the protein interactome.

In contrast to mitochondria and endoplasmic reticulum, peroxisomes are endowed with the ability to import oligomeric protein complexes (54–59). Combining this unique feature with the nanobody-delocalizing strategy opens up new perspectives for studying protein complexes using MS. Essentially, nanobodies can be equipped with a SKL peroxisomal targeting sequence that shuttles the nanobody and its target to the peroxisomal matrix (7). Target interaction partners could subsequently be identified using organellar proteomics. This technique implicates a subcellular fractionation step of the organelle of interest (e.g., peroxisomes), thus eliminating contaminating cytoplasmic proteins. Hence, sample complexity is compatible with the sensitivity of current mass spectrometers, allowing identification of low-abundance proteins (60). Moreover, as peroxisomal protein catalogs are available (61, 62), it is possible to discriminate true interaction partners from intrinsic peroxisomal protein. Of note, combining nanobody-induced delocalization with organellar proteomics has not been reported yet, implicating that one could encounter unexpected difficulties. The peroxisomal import machinery could possibly face difficulties in transporting large protein complexes, although successful import of 240 kDa tetrameric catalase has already been reported (54). Moreover, in order to obtain significant MS data, peroxisomes need to be isolated with high purity and adequate yields. Seeing that mammalian peroxisomes contribute to only 1–5% of the cell volume, this technique will probably require a substantial amount of cell material. Nevertheless, strategies to isolate pure and high yield peroxisomal fractions for organellar proteomics have been published (61).

As will be discussed in the next section, the nanobody-binding epitope could be used to identify “weak” spots in proteins, which offers opportunities for small molecule development. Hydrogen/deuterium exchange MS (HDX-MS) allows fast epitope characterization with small amounts of sample and has already frequently been used to characterize antibody epitopes (49–52). In brief, backbone amide hydrogens of the target protein are exchanged with deuterium. This process is subsequently repeated for the antibody-target protein complex. Antibody binding limits the accessibility of certain backbone hydrogens for deuterium exchange or alters the exchange rates. Consequently, the resulting MS fractionation patterns differ and allow delineation of the antibody epitope (49). We believe that this strategy could also successfully be exploited for nanobody epitope identification, although this has not been published yet.

## Nanobodies Used as Research Tool to Explore Protein Function

### Intracellular Nanobodies Interfering with Protein Function

Nanobodies represent a class of high-affinity inhibitors that, unlike RNAi, target proteins directly. They can be expressed in cells (intrabodies) with the purpose of knocking out (one or more) protein function(s), causing measurable effects (Table 3). The ultimate goal is to obtain better insight into otherwise poorly understood protein functions and signaling pathways. Moreover, this may represent a stepping stone toward rational drug development. For example, nanobodies were generated against β-catenin, a multi-functional protein, which has roles in cell–cell adhesion and transcriptional activation of Wnt responsive genes (31, 63). Mutations affecting the β-catenin/Wnt signaling pathway play a role in many diseases, including cancer. Newnham and coworkers developed a nanobody that specifically interfered with the transcriptional activating activity of β-catenin (63). This nanobody can enable further unraveling of the still intricate β-catenin/Wnt pathway. Analysis of the nanobody epitope could offer opportunities for development of small molecule inhibitors (63). Our lab obtained thoroughly characterized nanobodies against actin binding proteins cortactin, fascin, and L-plastin (64–67). We demonstrated their effects on actin bundling or branched actin polymerization, as well as their functional effects on podosome or invadopodia formation and dynamics, both specialized actin-rich membrane protrusions involved in (tumor) cell migration and invasion. In this way, we could sort out the precise contribution of specific protein domains in podosome or invadopodium formation and function (64–67). Our group also thoroughly characterized nanobodies against the DNA-binding domain of p53. We presented a nanobody that interferes with the transcriptional abilities of p53, while maintaining the functional architecture of p53 and even permitting p53 DNA-binding (68). Unlike other research tools, this nanobody allows targeting single functions of p53 with high precision (68). Nanobodies can also serve as elegant tools for the study and regulation of GPCR function. Different sets of nanobodies were developed against the model GPCR β_2_-adrenergic receptor (β_2_AR) (69–71). These nanobodies stabilize specific inactive or active conformations of the β_2_AR and thus are conformationally sensitive. All nanobodies recognize intracellular allosteric epitopes and can be expressed as intrabodies, without losing their preference for a distinctive GPCR conformation (70). Inhibitory nanobodies can, however, also be exploited extracellularly. The aforementioned ChemR23 nanobodies uniquely recognize ChemR23 GPCR and antagonize chemerin-induced receptor activation. As chemerin also binds other GPCRs, the nanobodies can be used to discriminate ChemR23-specific signaling from other chemerin-induced pathways (21).

**Table 3:** Overview of the different nanobody-based applications to explore protein function.

	Application	Strategy	Specifics	Reference
Explore protein function	Intracellular nanobodies interfering with protein function			(21, 31, 63–68, 70)

	Customize existing nanobodies by engineering	Delocalization		(7, 64)
		Converting non-invasive to invasive nanobodies		(9)
		Targeted protein degradation	*DeGradFP*	(72)
			Protein interference *(Protein-i)*	(73)
			Affinity-directed protein missile	(74)

	Nanobodies in X-ray crystallography			(69, 71, 75–77)

*Each application corresponds to the different sections in the main text and the strategies match the different paragraphs therein, which are whether or not further specified*.

### Customize Existing Nanobodies by Engineering

Existing (inhibitory and non-inhibitory) nanobodies can be engineered to expand their usefulness as a tool for investigating protein function (Table 3). Equipping nanobodies with an appropriate delocalization tag induces relocalization of the antigen–nanobody complex toward predetermined organelles and consequently, displaces the protein from where it is needed (7, 64). This can induce a loss-of-function, rather than a direct functional knockout. Correlating these findings with the use of untagged inhibitory nanobodies strengthens which protein functions are (not) important in particular pathways. For instance, we compared the effects of a fascin nanobody that disrupts fascin-mediated F-actin bundling on matrix metalloproteinase 9 (MMP-9) secretion, with its MOM-tagged counterpart (64). The latter nanobody is provided with a MOM delocalization tag and thus delocalizes endogenous fascin toward the outer mitochondrial membrane. Unlike untagged fascin nanobody, the MOM-fascin nanobody significantly reduced MMP-9 secretion, emphasizing a role for fascin in MMP-9 secretion independent of its actin-bundling activity (64).

Non-invasive intrabodies can also be engineered in such way that they can interfere with normal cell biology. As such, Jullien and colleagues transformed their H2A-H2B histon chromatibody into an invasive tool by coupling the nanobody to an E3 ubiquitin ligase (9). Expression of the fusion protein modifies the ubiquitin epigenetic landscape and dramatically distorts DNA double-strand break signaling and repair (9).

Our understanding of protein function has improved considerably by technologies that manipulate protein levels, such as RNAi or Morpholino antisense oligonucleotides. However, as these methods operate upstream of the protein level, they depend on the turnover rate of their target, thus resulting in limited depletion of long-lived proteins. Moreover, they frequently generate off-target effects (78, 79). To address these problems, systems directly acting on the protein level have been developed (80–82) and this is where also nanobodies can play a role.

**Figure 4:**
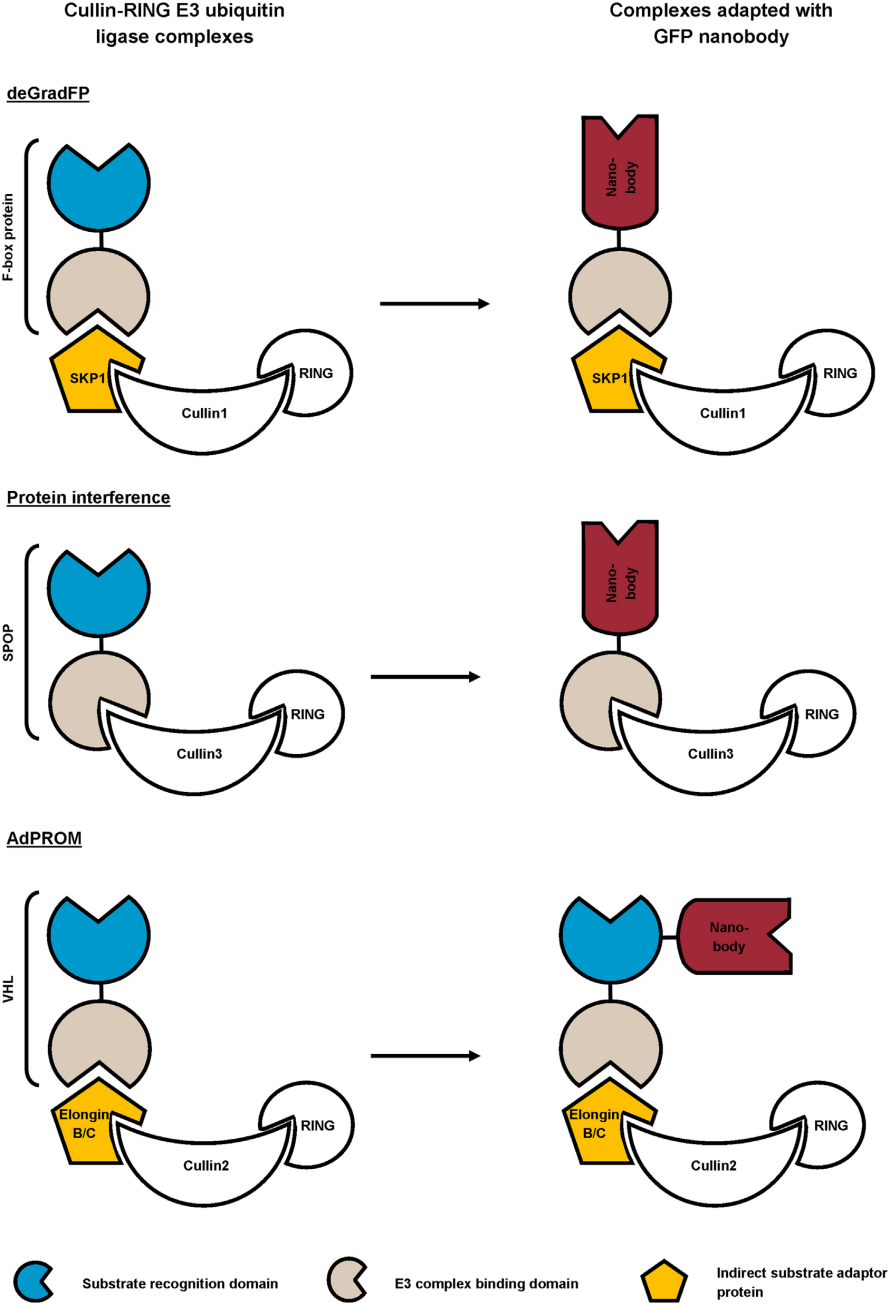
Schematic representation of reported strategies that combine the ubiquitin proteasome pathway with GFP nanobody for targeted degradation of GFP-fusion protein. In the deGradFP and Protein interference approach, the substrate recognition domain of the cullin-RING E3 ubiquitin ligase (CRL) complex is replaced by a high-affinity GFP nanobody. The affinity-directed protein missile (AdPROM) technique on the other hand, implies fusing the GFP nanobody with the substrate recognition domain of the complex.

Different research groups exploit the universal ubiquitin proteasome pathway in combination with high-affinity GFP nanobody for targeted protein degradation (72–74). To this end, they replaced the substrate recognition domain of cullin-RING E3 ubiquitin ligase (CRL) complexes with GFP nanobody (72, 73), or coupled a GFP nanobody to the recognition domain (74) (Figure 4). The CRL complexes are composed of a central cullin scaffold that interacts with an E2-recruiting RING protein *via* its C-terminal domain, and with a substrate adaptor protein *via* its N-terminus. The substrate adaptor protein mediates substrate specificity and recognizes its target directly (e.g., SPOP) or indirectly (e.g., SKP1 or Elongin B/C), the latter necessitating an additional adaptor protein (e.g., an F-box protein or VHL) for target binding. The CRL complexes ubiquitylate proteins and as such, mark them for degradation by the proteasome (83). In the deGradFP protocol, a GFP nanobody replaces the substrate recognition domain of an F-box protein, which in its turn recruits GFP-tagged proteins to the SKP1-cullin1 E3 ligase machinery (72). Conversely, the Protein interference (Protein-i) technique implies substituting the substrate recognition domain of adaptor SPOP with GFP nanobody. The GFP nanobody-SPOP fusion in its turn mediates GFP-fusion protein toward the cullin3 E3 ligase complex (73). Finally, the affinity-directed protein missile (AdPROM) approach fuses GFP nanobody with the C-terminal end of the VHL adaptor protein. This fusion protein mediates the association of GFP-tagged proteins with the Elongin B/C-cullin2 E3 complex for degradation. All techniques resulted in specific, fast ubiquitination and consequent proteasome-dependent degradation of GFP-fusion proteins in mammalian cells (72–74), *Drosophila* (72) and *Danio rerio* embryos (73). Compared to traditional RNAi, Protein-i even depleted proteins more rapidly and effectively. However, the Protein-i technique is currently limited to nuclear proteins, as the SPOP protein contains a nuclear localization signal (73). The deGradFP and AdPROM technologies can, however, be used for depletion of cytoplasmic proteins (72, 74). In summary, all three techniques hijack the same conserved pathway for targeted GFP-fusion protein degradation, but differ in range of action (nuclear and/or cytoplasmic) and GFP nanobody fusion (substitution of or fused with the substrate recognition domain). In theory, these techniques can be used for targeted degradation of virtually any (endogenous) protein, when replacing the GFP nanobody with a nanobody of choice.

Tang and coworkers developed a conditional system in which the stability of a nanobody depends upon the expression of its target (84). Therefore, they introduced destabilizing mutations in the nanobody FR, which could be transferred across different nanobodies (e.g., GFP, HIV-1 capsid protein CA, *Escherichia coli* dehydrofolate reductase nanobody, etc.) and even across nanobodies from different camelid species. Presence of the cognate antigen confers nanobody stability, whereas antigen absence results in proteasomal degradation of the nanobody and its associated tags. As such, it is possible to endow antigen-expressing subsets of cells with particular features. Tang and colleagues for instance exclusively labeled ACH-2 HIV-1 positive T-cells for flow cytometry (84). Therefore, they utilized destabilized chromobodies recognizing the HIV-1 capsid protein CA. Lack of CA expression in uninfected cells causes degradation of the destabilized chromobodies, consequently resulting in disappearance of fluorescence. Coupling a destabilized nanobody to Cas9 even allowed genome editing selectively in antigen-expressing cells using CRISPR/Cas (84). In theory, this technique can be combined with the aforementioned inhibitory nanobodies or with the deGradFP/Protein-i/AdPROM methods to interfere with protein function or target proteins for proteasomal degradation respectively, exclusively in cells expressing specific intracellular epitopes.

### Nanobodies in X-Ray Crystallography

Nanobodies also feature as a molecular lens in x-ray crystallography and thus can reveal molecular mechanisms or identify functionally important regions in a protein (Table 3). For instance, the crystal structure of a nanobody in complex with the serine protease urokinase-type plasminogen activator revealed valuable information on the mechanism by which peptide segments may act as strong protease inhibitors. The nanobody inserts its CDR3 loop into the active site of the protease in a substrate-like manner and becomes slowly cleaved. However, a rigid intra-loop interaction network which interconnects the putative scissile bond P1–P1′, holds the leaving group in place and favors reformation of the peptide bond over cleavage. The reaction reaches a cleavage-resynthesis equilibrium, thus rendering the nanobody into a strong inhibitor. Conversely, mutating specific amino acids in the CDR3 loop converts the nanobody to a strong substrate. These findings demonstrate the importance of the conformational rigidity of active-site binding peptide segments, when exploited as new protease inhibitors (75). On the other hand, Rudolph and colleagues reported the X-ray crystal structure of five nanobodies in complex with ricin toxin’s enzymatic subunit (RTA) (76, 77). The nanobodies all showed different ricin-neutralizing potencies (76, 77, 85). They identified RTA neutralizing hotspots which may prove useful in subunit vaccine development, seeing the low efficiency of current vaccination strategies (76, 77). Finally, when bound to their target, nanobodies can stabilize specific protein conformation and thus serve as chaperones in crystallography. The aforementioned β_2_AR nanobodies, binding different conformations of the GPCR, showed to be excellent chaperones in X-ray crystallography (69, 71) and NMR structural research (86), revealing the full allosteric potential of the β_2_AR.

## Conclusion and Perspectives

We have provided a brief overview of the various opportunities nanobodies offer in fundamental research, generally subdivided into the categories microscopy, protein–protein interactions, and protein function and we focused on how state-of-the-art engineering techniques can expand their versatility. Nanobodies feature small, stabe (intracellularly), and soluble high-affinity targeting moieties that can easily be produced. Moreover, it is possible to engineer nanobodies in such a way that they display a desired function or set of functions (e.g., fluorescence, delocalization, degradation, etc.), without interfering with its binding characteristics. Hence, they are highly adaptable. These favorable characteristics stimulated their use as research tools in diverse aspects of fundamental research. Undoubtedly, in future years, new applications will continue to surface.

## Author Contributions

EB and JG wrote the manuscript. All authors reviewed the manuscript.

## Conflict of Interest Statement

The authors declare that the research was conducted in the absence of any commercial or financial relationships that could be construed as a potential conflict of interest. The reviewer, GH, and handling editor declared their shared affiliation, and the handling editor states that the process nevertheless met the standards of a fair and objective review.
